# Voriconazole-associated liver injury: clinical risk factor identification and predictive nomogram construction

**DOI:** 10.3389/fphar.2025.1688711

**Published:** 2026-01-05

**Authors:** Dongge Feng, Yiyi Ma, Xiaoyi Liu, Si Tang, Shijian Xiang, Jinghao Wang, Shasha Li

**Affiliations:** 1 Department of Pharmacy, The First Affiliated Hospital of Jinan University, Guangzhou, China; 2 School of Pharmacy, Jinan University, Guangzhou, China; 3 Department of Pharmacy, The Seventh Affiliated Hospital, Sun Yat-sen University, Shenzhen, China

**Keywords:** voriconazole, nomogram, drug-induced liver injury, predictive model, real-world study

## Abstract

**Objective:**

Investigate the high risk factors contributed to voriconazole-induced liver injury and develop a predictive nomogram for voriconazole-induced liver injury risk, thereby optimizing the clinical medication safety.

**Methods:**

This observational study retrospectively analyzed the electronic health records of hospitalized patients who received voriconazole treatment or prophylaxis at a tertiary hospital. Patients who met the inclusion and exclusion criteria were enrolled between June 2020 and June 2024, and their general biological data were collected. The diagnosis and severity grading of voriconazole-associated liver injury were determined according to the diagnostic and treatment guidelines for drug-induced liver injury. Patients were categorized into liver injury and non-injury groups based on the results of post-treatment liver function tests. Associations between baseline characteristics and liver injury were analyzed using non-parametric and chi-squared tests. To avoid omitting potentially significant factors, variables demonstrating *P* ≤ 0.1 in univariate screening and retained through least absolute shrinkage and selection operator (LASSO) regression underwent multivariate logistic regression to identify independent predictors. The nomogram was developed and internally validated through receiver operating characteristic (ROC) analysis, calibration curves, and decision curve analysis (DCA).

**Results:**

Among the 1,964 patients screened, 1,196 were excluded, leaving 768 patients included in the statistical analysis. Liver injury occurred in 95 patients, resulting in an incidence rate of 12.4%. Multivariable logistic regression analysis demonstrated that total cholesterol (TC) (OR = 1.893, *P* < 0.01), concomitant use of glucocorticoids (OR = 1.861, *P* = 0.041), ezetimibe (OR = 7.453, *P* = 0.047), and caspofungin (OR = 2.485, *P* = 0.032). Patients with TC levels exceeding 4.485 mmol/L exhibited a significantly elevated risk of liver injury. ROC analysis revealed an area under the curve (AUC) of 0.728 (95% CI: 0.66–0.797), with a sensitivity of 0.661 and specificity of 0.722. Internal validation indicated good discrimination and calibration, with predicted probabilities closely aligned with actual outcomes. The decision curve analysis suggested a substantial net clinical benefit. These findings were subsequently validated in a test cohort.

**Conclusion:**

Concomitant use of ezetimibe, caspofungin, glucocorticoids, and TC levels exceeding 4.485 mmol/L are independent risk factors for voriconazole-induced liver injury. The developed nomogram model offers clinically meaningful predictions for drug-induced liver injury risk, facilitating the optimization of voriconazole therapy safety.

## 1 Introduction

Invasive Fungal Infections (IFD) typically occur in individuals with severely compromised immune systems, impaired host barrier functions, or exposure to high-risk environments (Taccone et al., 2015; Perreault et al., 2019; Boutin et al., 2024). Voriconazole, a broad-spectrum triazole antifungal, is extensively employed in treat severe IFD (Patterson et al., 2016; Yang et al., 2022). However, as the clinical use of voriconazole has increased, adverse effects such as drug-induced liver injury (DILI), neurotoxicity, visual disturbances, and gastrointestinal reactions have become more prevalent (Jin et al., 2016). Among these, DILI is the most common and severe adverse reaction, and it is a major reason for treatment discontinuation. DILI can be categorized into non-idiosyncratic and idiosyncratic types. Non-idiosyncratic liver injury is dose- or duration-dependent, with a short latency period, and is attributed to direct hepatotoxicity from drugs or their metabolites (Tiwari et al., 2025). In contrast, voriconazole-associated DILI is primarily idiosyncratic and linked to individual genetic and metabolic variations, as well as immune responses, with no clear dose dependency. Conventional animal toxicology studies often fail to accurately predict clinical toxicity risks in such cases (Tiwari et al., 2025; Du et al., 2023). Recent studies have reported a DILI incidence of 12.9%–32.45% during voriconazole therapy (Perreault et al., 2019; Shen et al., 2022; Hanai et al., 2023; Wang et al., 2022; Zhou et al., 2022), highlighting it as a critical safety concern in clinical practice. Early identification and timely intervention can significantly mitigate these risks; however, there remains a lack of specific predictive tools for voriconazole-related liver injury.

Voriconazole-induced DILI has been linked to mitochondrial dysfunction, oxidative stress, and disrupted bile acid homeostasis (Watkins, 2019; Wu et al., 2020). Critically, the specific targets responsible for the induction of liver injury remain unidentified. Therefore, it is crucial to elucidate the risk factors associated with voriconazole-induced DILI and to predict its occurrence following voriconazole administration. Previous studies have employed machine learning algorithms to develop predictive models. These models utilized clinical features to forecast voriconazole trough plasma concentrations, aiding in liver injury risk assessment (Cheng et al., 2023). Furthermore, comparisons among various machine learning models revealed that the logistic regression model exhibited superior performance in predicting voriconazole-related hepatotoxicity (Ma et al., 2023). Despite the development of these models, limitations persist, including inadequate interpretability, a strong dependence on clinical features, and a lack of intuitive visualization of the risk assessment process in clinical applications.

This study delineates key predictors of voriconazole-associated DILI and to develop a nomogram-based risk prediction model utilizing the identified independent risk factors. This approach addresses the limitations of existing research by enhancing model interpretability and clinical utility, thereby facilitating individualized dosing regimens and improving medication safety.

## 2 Population and methods

### 2.1 Patients

This study retrospectively analyzed the electronic health records of patients who used voriconazole for therapeutic and prophylactic indications of invasive fungal diseases at The First Affiliated Hospital of Jinan University from June 2020 to June 2024.

Inclusion criteria: age ≥18 years, who received voriconazole for the treatment or prophylaxis of IFD, underwent liver function biochemical tests during voriconazole therapy, and had all baseline liver function indices (prior to voriconazole initiation) within the upper limit of normal (ULN).

Exclusion criteria: age <18 years; presence of liver injury caused by viral hepatitis, autoimmune hepatitis, hepatic failure, alcohol-related liver diseases, metabolic-associated and fatty liver diseases, drug and toxin-induced liver injury, biliary tract diseases, or hepatocellular carcinoma; absence of baseline liver function tests performed before voriconazole initiation; and baseline liver indices (alanine aminotransferase (ALT), aspartate aminotransferase (AST), gamma-glutamyl transferase (γ-GGT), total bilirubin (T-BiL), and alkaline phosphatase (ALP)) exceeding 1×ULN).

### 2.2 Methods

The electronic health records of enrolled patients were retrospectively extracted from the hospital’s integrated information system. The data collection encompassed demographic characteristics, therapeutic regimens, serial laboratory parameters (specifically liver function profiles), documented adverse drug reactions (ADRs), and clinical outcomes.

Severity Grading of Acute DILI: Following diagnosis of acute DILI, severity was classified into four grades based on criteria established by the International DILI Expert Working Group: Grade 1 (Mild): ALT ≥ 5×ULN or ALP ≥ 2×ULN with total bilirubin (T-BiL) < 2×ULN. Grade 2 (Moderate): ALT ≥ 5×ULN or ALP ≥ 2×ULN with T-BiL ≥ 2×ULN, or symptomatic hepatitis. Grade 3 (Severe): ALT ≥ 5×ULN or ALP ≥ 2×ULN with T-BiL ≥ 2×ULN, plus one of the following: INR ≥ 1.5, ascites/hepatic encephalopathy, disease duration <26 weeks (without pre-existing cirrhosis), or DILI-induced extrahepatic organ failure. Grade 4 (Fatal): DILI-related death or the necessity for liver transplantation for survival (Meunier and Larrey, 2023).

The causal relationship between liver injury and voriconazole use was evaluated using the Roussel Uclaf Causality Assessment Method (RUCAM) (Danan and Teschke, 2015). Patients with a RUCAM score of ≥6 were classified as having highly probable or probable voriconazole-induced drug-induced liver injury. Those with a RUCAM score of <6 were excluded from further risk factor analyses due to insufficient evidence to reliably attribute liver injury to voriconazole therapy.

Diagnosis of Liver Injury Clinical Types: The clinical type of liver injury is classified using the R-value, which is calculated as the ratio of [ALT/(ALT ULN)] to [ALP/(ALP ULN)]. The categories are defined as follows: Hepatocellular Injury (R ≥ 5): Characterized by a predominant elevation of ALT or AST. Cholestatic Injury (R ≤ 2): Defined by elevated ALP and/or γ-GGT. Mixed Pattern (2 < R < 5): Exhibits combined hepatocellular and cholestatic biochemical features (Aithal et al., 2011; Colombo, 2020; Andrade et al., 2019; Freites-Martinez et al., 2021). Classification by Disease Course: Acute Liver Injury: Resolves within 6 months of onset. Chronic Liver Injury: Persists for more than 6 months with unresolved biochemical or histological abnormalities.

### 2.3 Statistical analysis

Patient data were systematically entered and organized using Microsoft Excel 2020. After thorough verification, the dataset was imported into SPSS 27.0 (IBM Corp.) for comprehensive statistical analysis. Normality for all continuous variables was tested with the Kolmogorov-Smirnov test. For non-normally distributed data, between-group comparisons were performed using the Mann-Whitney U test, with data reported as median (interquartile range). Normally distributed continuous variables are presented as mean ± standard deviation and compared via Student's t-test. Categorical variables underwent χ^2^ test or Fisher’s exact test when the expected frequencies were less than.

### 2.4 Feature selection and model development

Least absolute shrinkage and selection operator (LASSO) regression was applied to identify significant risk factors for voriconazole-associated drug-induced liver injury by shrinking the coefficients of less relevant predictors to zero. To avoid omitting potential risk factors, candidate variables selected by both univariate analysis (*P* ≤ 0.1) and LASSO regression were included in a final multivariable logistic regression model to define independent risk factors (Hanai et al., 2023; Ranganathan et al., 2017).

To ensure a robust and unbiased model evaluation, random stratified sampling was employed to partition the dataset into training and testing sets in a 7:3 ratio. The training set was utilized for model development, while the testing set served for model performance evaluation. The dataset was stratified based on the binary outcome of liver injury occurrence (yes/no). This approach ensured a comparable prevalence of positive events (liver injury = 1) across the two resulting subsets. Within each stratum, a simple random sampling procedure was implemented using a fixed random seed [set.seed (42)] to guarantee the reproducibility of the data split. The class imbalance between positive cases (hepatotoxicity) and negative cases was addressed using the Synthetic Minority Oversampling Technique. A nomogram model was developed using R software (version 4.4.2) to visualize the risk prediction algorithm. The model’s performance was evaluated by its area under the curve (AUC), with internal validation via 1,000 bootstrap resamples to correct for overfitting. Its clinical utility was further quantified using decision curve analysis (DCA).

## 3 Results

### 3.1 Patient characteristics

**FIGURE 1:**
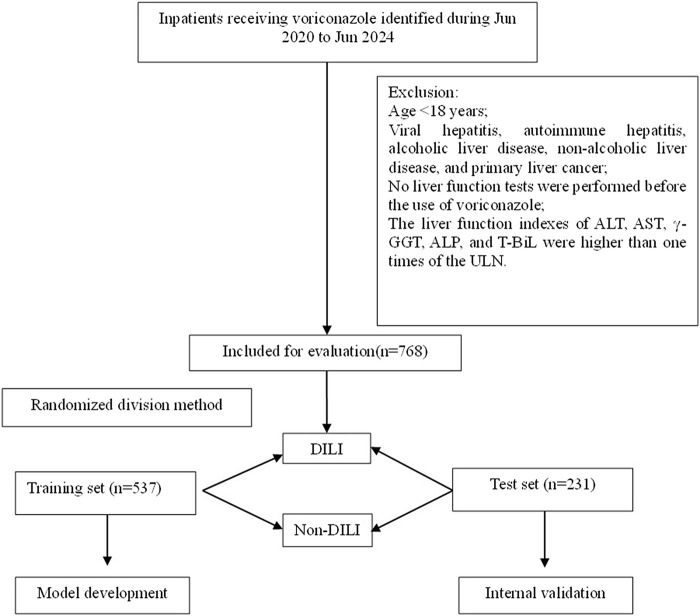
Flowchart of patient inclusion and exclusion.

A total of 768 patients who were exposed to voriconazole between June 2020 and June 2024 were retrospectively analyzed after applying the inclusion and exclusion criteria outlined in Figure 1. The cohort comprised 487 males (63.4%) and 281 females (36.6%), with 39 patients (5.1%) reported a history of alcohol use and 91 patients (11.8%) had a smoking history.

The majority of patients were from the hematology department (n = 387, 50.4%), followed by the respiratory department (n = 120, 15.6%) and the ICU (n = 108, 14.1%). The identified fungal pathogens included *Aspergillus* spp. (n = 152, 19.8%), *Candida* spp. (n = 122, 15.9%), and other fungi (n = 63, 8.2%), such as fungal spores, *Lichtheimia corymbifera*, *Trichosporon asahii*, and *Pneumocystis jirovecii*.

Infections predominantly involved the respiratory system (n = 365, 47.5%), followed by the urinary system (n = 38, 4.9%). Following voriconazole therapy, 508 patients (66.1%) demonstrated clinical improvement. Detailed results are presented in Supplementary Table S1.

### 3.2 Adverse drug reactions, management strategies, and clinical outcomes of voriconazole therapy

A total of 213 patients (27.7%) experienced ADRs during voriconazole therapy, with a median onset time of 4 days. The most common ADR was DILI (n = 95, 12.4%), followed by neurotoxicity (n = 25, 3.3%), visual disturbances (n = 9, 1.2%), and other reactions (n = 24, 3.1%), which included rash, flushing, transient fever, tinnitus, diarrhea, elevated serum creatinine, and palpitations. Among 95 patients with voriconazole-induced liver injury, the severity was graded as 1 in 44 (46.3%), 2 in 30 (31.6%), 3 in 18 (18.9%), and 4 in 3 (3.2%) patients. According to clinical phenotype, the injury was classified as hepatocellular in 33 (34.7%), cholestatic in 42 (44.2%), and mixed in 20 (21.1%) patients.

Management strategies involved switching from oral voriconazole tablets to intravenous formulations for patients experiencing intractable nausea or vomiting, administering hepatoprotective agents (e.g., glutathione, polyene phosphatidylcholine) to those with abnormal liver function, adding mecobalamin for mild neurotoxicity, and discontinuing voriconazole in cases of severe ADRs. These interventions led to symptom resolution or significant improvement in 89.2% of affected patients (n = 190/213). Detailed results are presented in Supplementary Table S1.

### 3.3 High-risk factors of voriconazole DILI

**FIGURE 2:**
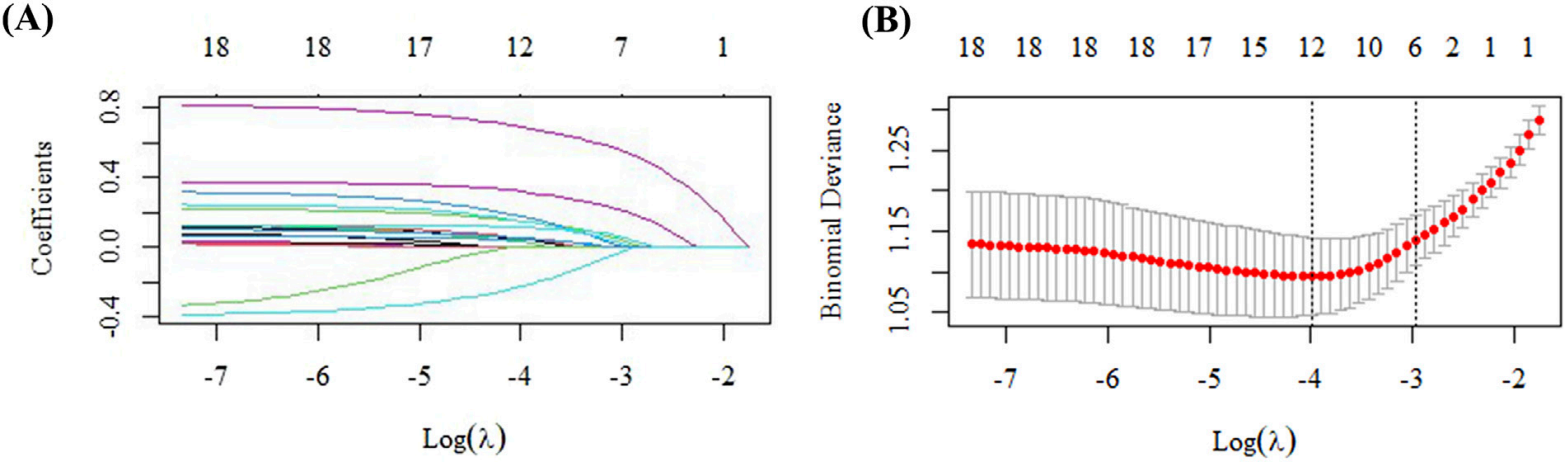
Variable selection via LASSO regression in the training cohort. **(A)** Identification of the optimal penalty parameter (λ) through 10-fold cross-validation. **(B)** Profile of the LASSO coefficients for all clinical features across different λ values.

The study cohort of 768 patients was randomly divided into a training set (n = 537, 70%) and a test set (n = 231, 30%) using a 7:3 allocation ratio. Clinical data from the training set underwent univariate analysis and LASSO regression analysis, followed by logistic regression to identify independent risk factors for voriconazole-induced liver injury. The results of these analyses were summarized in Table 1; Figure 2.

**TABLE 1:** Univariate Logistic Regression: Voriconazole and Liver Injury in training and test Sets.

Risk factor	Training set (n = 537)		P-value	Test set (n = 231)		P-value
	DILI (62)	Non-DILI (475)		DILI (33)	Non-DILI (198)	
Basic characteristics						
Age	61 (50.5,70)	62 (48,72)	0.638	66 (42.5,81)	66 (50,76)	0.776
Gender (female)	42 (67.7)	303 (63.8)	0.541	21 (63.6)	121 (61.1)	0.783
Alcohol	4 (6.5)	24 (5.1)	0.641	1 (3.0)	10 (5.1)	0.594
Smoking	7 (11.3)	63 (13.3)	0.664	4 (12.1)	17 (8.6)	0.528
Length of hospital stay	27 (14.5,46.5)	23 (13,38)	0.244	23 (15,43)	25 (14,35)	0.853
Comorbidities						
Hypoproteinemia	21 (33.9)	136 (28.6)	0.394	12 (36.4)	59 (29.8)	0.449
Malignant tumor	6 (9.7)	51 (10.7)	0.799	5 (15.2)	18 (9.1)	0.308
Hematologic malignancies	24 (38.7)	186 (39.2)	0.946	12 (36.4)	79 (39.9)	0.700
Septic shock	11 (17.7)	48 (10.1)	0.071	10 (30.3)	17 (8.6)	0.001
Hyperuricemia	3 (4.8)	38 (8.0)	0.351	0 (0)	11 (5.6)	0.062
Hyperlipidemia	1 (1.6)	23 (4.8)	0.191	2 (6.1)	10 (5.1)	0.813
Anemia	10 (16.1)	51 (10.7)	0.208	7 (21.2)	19 (9.6)	0.071
COPD	2 (3.2)	24 (5.1)	0.506	1 (3.0)	7 (3.5)	0.881
Hypertension	17 (27.4)	151 (31.8)	0.485	12 (36.4)	61 (30.8)	0.525
Diabetes mellitus	16 (25.8)	89 (18.7)	0.187	10 (30.3)	31 (15.7)	0.041
Pretreatment laboratory parameters for voriconazole						
Body temperature (°C)	36.95 (36.7,37.8)	36.9 (36.5,37.6)	0.323	37 (36.8,38.1)	36.9 (36.5,37.6)	0.254
WBC (×10^9^/L)	8.01 (3.72,12.56)	6.15 (2.88,9.33)	0.089	9.18 (4.03,12.47)	6.25 (2.77,8.93)	0.017
Serum creatinine (μmol/L)	83 (64.83,132.29)	78.3 (56.9,132.29)	0.225	107.7 (63.5,172.6)	78 (57.88,132.29)	0.111
Urea (mmol/L)	8.69 (5.69,12.99)	6.69 (4.61,12.4)	0.127	11.61 (5.55,20.69)	7.38 (4.69,11.61)	0.054
PCT (ng/mL)	1.32 (0.27,5.25)	0.48 (0.14,5.25)	0.048	1.04 (0.31,6.06)	0.46 (0.14,5.25)	0.034
γ-GGT (U/L)	32.55 (24.75,47)	32.55 (25,35)	0.122	37 (29,47.5)	32.51 (25,37)	0.02
ALT (U/L)	17 (11,23.5)	20 (12,29)	0.433	20 (14,30.5)	17.5 (12,24.25)	0.102
AST (U/L)	20.5 (15,27)	23 (16,30)	0.368	26 (20.5,31)	22 (17,28.25)	0.046
ALB (g/L)	33.05 (28.7,35.73)	33.57 (30,38.2)	0.118	32.4 (25.6,37.35)	33.57 (29.78,37.65)	0.115
D-BiL (μmol/L)	3.46 (2.98,4.53)	3.46 (2.5,3.9)	0.266	2.9 (2.3,3.48)	3.46 (2.48,3.73)	0.155
T-BiL (μmol/L)	10.2 (7.9,12)	11.27 (8.4,11.5)	0.266	10.8 (6.3,14.3)	11.27 (8.7,11.27)	0.557
TC (mmol/L)	4.96 (4.06,5.64)	3.94 (3.22,4.61)	<0.001	5.11 (4.06,5.87)	3.84 (2.94,4.59)	<0.001
TG (mmol/L)	1.51 (1.07,2.04)	1.54 (0.99,2.12)	0.771	1.78 (1.15,2.23)	1.37 (0.91,2.0)	0.238
HDL (mmol/L)	0.98 (0.71,1.21)	0.93 (0.7,1.21)	0.901	0.98 (0.64,1.22)	0.915 (0.67,1.19)	0.499
LDL (mmol/L)	2.02 (1.41,2.63)	2.17 (1.6,2.76)	0.266	2.01 (1.62,2.55)	2.05 (1.44,2.53)	0.847
Concomitant medications - antibiotics						
Carbapenems	29 (46.8)	175 (36.8)	0.130	16 (48.5)	80 (40.4)	0.383
Beta-lactam/enzyme inhibitor combinations	19 (30.6)	190 (40)	0.308	11 (33.3)	83 (41.9)	0.353
Fluoroquinolones	21 (33.9)	137 (28.8)	0.414	11 (33.3)	71 (35.9)	0.779
Polypeptides	15 (24.2)	109 (22.9)	0.827	7 (21.2)	37 (18.7)	0.732
Cephalosporins	6 (9.7)	35 (7.4)	0.697	3 (9.1)	17 (8.6)	0.924
Aminoglycosides	3 (4.8)	14 (2.9)	0.452	0 (0)	6 (3.0)	0.171
Macrolides	1 (1.6)	6 (1.3)	0.825	0 (0)	3 (1.5)	0.334
Tetracyclines	7 (11.3)	48 (10.1)	0.772	3 (9.1)	16 (8.1)	0.847
Linezolid	14 (22.6)	81 (17.1)	0.283	6 (18.2)	36 (18.2)	1.0
Sulfamethoxazole	9 (14.5)	30 (6.3)	0.034	3 (9.1)	12 (6.1)	0.532
Other medications						
Glucocorticoids	24 (38.7)	127 (26.7)	0.049	11 (33.3)	49 (24.7)	0.298
Statins	6 (9.7)	45 (9.5)	0.959	5 (15.2)	19 (9.6)	0.356
Fenofibrate	2 (3.2)	13 (2.7)	0.83	0 (0)	3 (1.5)	0.334
Caspofungin	10 (16.1)	36 (7.6)	0.024	4 (12.1)	15 (7.6)	0.379
Cytarabine	4 (6.5)	33 (6.9)	0.884	4 (12.1)	11 (5.6)	0.193
Calcium channel blockers	8 (12.9)	45 (9.5)	0.394	5 (15.2)	23 (11.6)	0.575
β-adrenergic antagonist	4 (6.5)	78 (16.4)	0.04	6 (18.2)	32 (16.2)	0.772
Ezetimibe	2 (3.2)	3 (0.6)	0.045	0 (0)	2 (1.0)	0.431
Metformin	2 (3.2)	17 (3.6)	0.886	1 (3.0)	3 (1.5)	0.569
Mycophenolate mofetil	3 (4.8)	10 (2.1)	0.236	0 (0)	6 (3.0)	0.171
Idarubicin	1 (1.6)	13 (2.7)	0.578	0 (0)	7 (3.5)	0.139
Vincristine	1 (1.6)	3 (0.6)	0.454	0 (0)	3 (1.5)	0.334
Rabeprazole	4 (6.5)	11 (2.3)	0.063	2 (6.1)	3 (1.5)	0.153
Omeprazole	1 (1.6)	1 (0.2)	0.088	0 (0)	0 (0)	
Ilaprazole	2 (3.2)	6 (1.3)	0.287	2 (6.1)	3 (1.5)	0.153
Pantoprazole	0 (0)	2 (0.4)	0.483	1 (3.0)	0 (0)	0.048
Bromhexine	4 (6.5)	12 (2.5)	0.087	1 (3.0)	7 (3.5)	0.881
Salbutamol	1 (1.6)	1 (0.2)	0.088	1 (3.0)	1 (0.5)	0.229
Montelukast sodium	2 (3.2)	4 (0.8)	0.093	3 (9.1)	2 (1.0)	0.016
Terbutaline	8 (12.9)	24 (5.1)	0.028	5 (15.2)	8 (4.0)	0.026

Abbreviations: COPD, chronic obstructive pulmonary disease; WBC, white blood cell count; GFR, glomerular filtration rate; PCT, procalcitonin; ALT, alanine aminotransferase; AST, aspartate aminotransferase; ALP, alkaline phosphatase; ALB, albumin; T-BiL, total bilirubin; D-BiL, direct bilirubin; γ-GGT, Gamma-Glutamyl Transferase; TC, total cholesterol; TG, triglycerides; HDL, High-Density Lipoprotein; LDL, Low-Density Lipoprotein. Categorical variables were analyzed using the χ^2^ test, Fisher’s exact test, or Wilcoxon rank-sum test to estimate *P*-values, while continuous variables were evaluated via the Mann-Whitney *U* test as appropriate.

In the training set (n = 537), patients were stratified into two groups: DILI (n = 62) and Non-DILI (n = 475). Analysis of the training set indicated that the occurrence of voriconazole-induced DILI was marginally associated with septic shock (*P* = 0.071). Significant associations were found with the concomitant use of sulfamethoxazole (*P* = 0.034), glucocorticoids (*P* = 0.049), caspofungin (*P* = 0.024), ezetimibe (*P* = 0.045), and terbutaline (*P* = 0.028). Additionally, borderline trends were noted for rabeprazole (*P* = 0.063), omeprazole (*P* = 0.088), bromhexine (*P* = 0.087), salbutamol (*P* = 0.088), and montelukast (*P* = 0.093). Laboratory parameters associated with DILI risk included an elevated white blood cell count (*P* = 0.089), procalcitonin (PCT) (*P* = 0.048), and total cholesterol (TC) (*P* < 0.05). These key indices were subsequently entered into a LASSO regression model, and variables with non-zero coefficients were retained. At λ.1se = 0.0367, the selected variables included sulfamethoxazole, caspofungin, glucocorticoids, β-blockers, bromhexine, montelukast sodium, terbutaline, ezetimibe, PCT, white blood cell count, and total cholesterol, which were then incorporated into a logistic regression to establish a new prediction model (Supplementary Table S2).

The final model identified the concomitant use of glucocorticoids, ezetimibe, caspofungin, and elevated TC levels as independent risk factors for voriconazole-induced DILI. Detailed regression coefficients, odds ratios, and corresponding *P*-values are presented in Table 2.

**TABLE 2:** Multivariate analysis of the risk factors for voriconazole-induced liver injury.

Risk factor	B	SE	Wald	*Exp(B)*	95% CI	*P* value
Glucocorticoids	0.62	0.31	4.57	1.86	1.03–3.38	0.041
Ezetimibe	2.01	0.57	3.93	7.45	1.02–54.3	0.047
Caspofungin	0.91	0.42	4.61	2.49	1.08–5.71	0.032
TC	0.64	0.12	29.84	1.89	1.51–2.38	<0.01

### 3.4 Prediction model development

**FIGURE 3:**
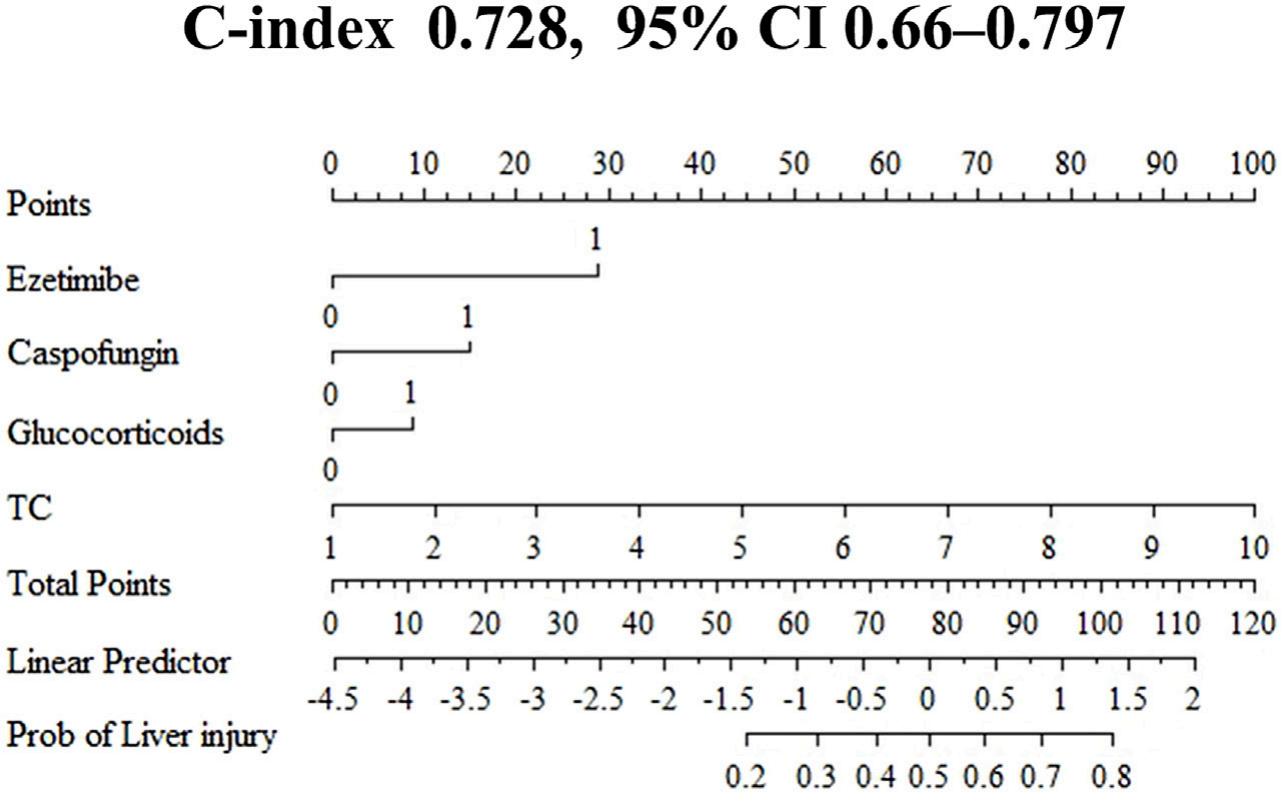
Nomogram for predicting the risk of voriconazole-induced liver injury. All binary clinical variables are coded as 0 or 1, where 0 represents “No” (or absence) and 1 represents “Yes” (or presence). Specifically for the variables “ezetimibe,” “caspofungin,” and “glucocorticoids,” a value of 0 indicates “not used” and 1 indicates “used.” TC, total cholesterol (mmol/L). The “Points” scale assigns a score for each variable level; the total points correspond to the value on the “Linear Predictor” axis, which is then converted to the estimated risk of liver injury (“Risk” axis).

Based on the high risk factors identified through logistic regression, a nomogram for predicting voriconazole-induced DILI was constructed using R 4.4.2 (Figure 3). Each risk factor-ezetimibe use, glucocorticoid use, caspofungin use, and TC levels—was assigned a score ranging from 0 to 100 points, proportional to its contribution to the outcome. Individual scores were calculated based on the value of each predictor, and the total score was converted into a probability of DILI occurrence through a predefined functional relationship. Higher total scores correlated with an increased risk of DILI.

### 3.5 Prediction model evaluation

**FIGURE 4:**
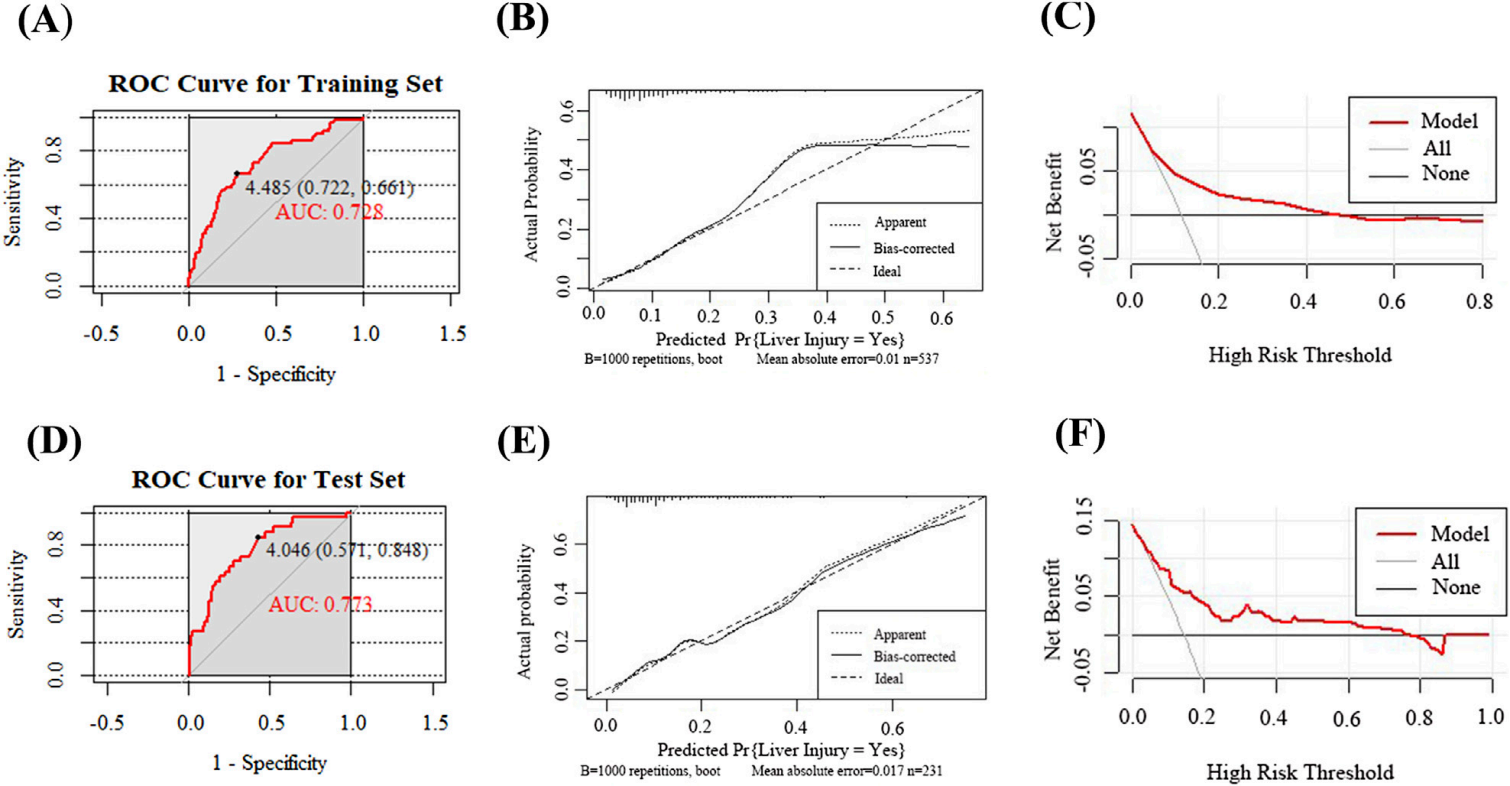
Evaluation and validation of the voriconazole-induced liver injury prediction model. **(A)** ROC curve of the model in the training set; **(B)** Calibration curve in the training set; **(C)** DCA in the training set; **(D)** ROC curve in the test set; **(E)** Calibration curve in the test set; **(F)** DCA in the test set.

The ROC curve for the training set (Figure 4A) demonstrated an AUC of 0.728 (95% CI: 0.660–0.797), with a sensitivity of 0.661, specificity of 0.722, and an optimal cut off value for TC of 4.485 mmol/L to stratify the risk of DILI.

The calibration of the nomogram was evaluated to assess the accuracy of the predicted probabilities for clinical outcomes. The calibration curve illustrated the concordance between the predicted and observed event rates. Following internal validation with 1,000 bootstrap resamples, the calibration curve of the training set demonstrated close alignment with the ideal line (45° reference), indicating good calibration performance with minimal deviation between the predicted and observed results (Figure 4B).

The model’s clinical value was assessed via DCA, as illustrated in Figure 4C. The black line, representing the “None” strategy, indicates the net benefit when no patients are diagnosed with DILI, while the light gray line, corresponding to the “All” strategy, reflects the net benefit assuming all patients are diagnosed with DILI. Within the threshold probability range of 20%–50% (0.2–0.5), the nomogram, depicted by the red curve, demonstrated a higher net benefit compared to both the “All” and “None” strategies. This finding suggests that the nomogram is optimal for guiding clinical decisions in patients with moderate-risk thresholds. At lower threshold probabilities, the model may also assist in identifying individuals who require intervention, thereby enhancing the overall net clinical benefit.

### 3.6 Prediction model validation

Following the successful development of the prediction model, the nomogram underwent internal validation using the test set. ROC curve analysis (Figure 4D) revealed an AUC of 0.773 (95% CI: 0.689–0.857), with a sensitivity of 0.848 and specificity of 0.571, indicating a robust discriminative ability. The calibration curve for the test set displayed close alignment with the ideal line, reflecting minimal deviation between predicted and observed outcomes, thereby confirming good calibration performance (Figure 4E). DCA further demonstrated that the model provided substantial net clinical benefit at low-risk thresholds; however, its utility diminished progressively with increasing threshold probabilities, suggesting limited clinical applicability in high-risk scenarios (Figure 4F).

## 4 Discussion

Voriconazole, this triazole-class antifungal is indicated for systemic IFD (Boutin et al., 2024; Allegra et al., 2020). However, the increasing clinical use of voriconazole has been associated with a rise in reported ADRs, including DILI, neurotoxicity, and visual disturbances. Idiosyncratic liver injury is one of the most common and severe complications, serving as a leading cause for the discontinuation of drug development in clinical trials and restrictions on post-marketing use (Yang et al., 2022; Jin et al., 2016). Notably, hepatotoxicity accounted for 32% of drug withdrawals between 1975 and 2007 (Stevens and Baker, 2009), highlighting its significant impact on drug safety and regulatory decision-making (Andrade et al., 2019). This retrospective study analyzed the risk factors for ADRs in patients receiving voriconazole treatment or prophylaxis. The overall incidence of ADRs was 27.7%, with DILI occurring in 12.4% of cases. Importantly, the observed DILI rate in this Chinese cohort exceeds the 1%–10% incidence of hepatic dysfunction reported in Western populations (Den Hollander et al., 2006; Shehab et al., 2007). This discrepancy may be attributed to genetic polymorphisms in CYP2C19, a key enzyme involved in voriconazole metabolism. Asian populations exhibit a higher prevalence of CYP2C19 poor metabolizers, resulting in a prolonged voriconazole half-life, delayed attainment of steady-state concentrations, and altered plasma levels (Shimizu et al., 2003; Chen et al., 2018). The concomitant use of CYP inducers (e.g., rifampicin, phenytoin) reduces voriconazole plasma concentrations, whereas coadministration with CYP inhibitors (e.g., omeprazole) or competitive substrates (e.g., warfarin) may elevate drug exposure, thereby increasing the risk of hepatotoxicity (Schulz et al., 2019).

In hospitalized patients, ADRs include hepatic dysfunction, gastrointestinal disturbances, neurotoxicity, delirium, and visual impairments, which typically emerge within 2–8 days of therapy initiation. Early hepatic injury is characterized by elevated γ-GGT levels, followed by subsequent increases in transaminase levels (ALT/AST). The increase in γ-GGT may serve as an early warning signal for hepatotoxicity, while transaminase elevation reflects progressive hepatocellular damage.

DILI is typically attributed to three primary mechanisms: mitochondrial dysfunction, disruption of bile acid homeostasis and oxidative stress (Watkins, 2019; Wu et al., 2020). This study identified TC and the concomitant use of ezetimibe, caspofungin, and glucocorticoids as independent risk factors for voriconazole-induced DILI. Prior research indicates that voriconazole therapy increases serum triglycerides and TC to 4.55-fold and 3.31-fold above baseline levels, respectively, with dyslipidemia positively correlating with voriconazole plasma concentrations (Wu et al., 2021). TC, a biomarker of lipid metabolism, may reflect hepatic lipid accumulation, potentially impairing the metabolic capacity of voriconazole and increasing susceptibility to DILI. Excessive cholesterol reduces membrane fluidity, disrupts microdomains, and alters protein function, culminating in cellular dysfunction and death (Song et al., 2021). Elevated TC levels may also exacerbate hepatotoxicity through chronic inflammation and oxidative stress, both of which are known contributors to drug-induced liver injury. Excessive cholesterol induces hepatocyte damage primarily via mitochondrial dysfunction and Kupffer cell foaming. In mitochondria, it increases membrane rigidity, disrupts protein function, and depletes glutathione, promoting reactive oxygen species (ROS) production (Marí et al., 2020). Concurrently, cholesterol crystal formation triggers hepatocyte necrosis and inflammasome activation in Kupffer cells, driving inflammation and liver injury (Ioannou et al., 2017).

This study identifies ezetimibe as a significant risk factor for voriconazole-induced liver injury. As an intestinal cholesterol absorption inhibitor, ezetimibe may alter bile composition or flow and inhibit drug transporters (e.g., P-glycoprotein or Organic Anion Transporting Polypeptide), potentially elevating voriconazole plasma concentrations and exacerbating hepatotoxicity (Couture and Lamarche, 2013). Although ezetimibe is primarily metabolized via glucuronidation, its partial metabolism still depends on CYP3A4. Concurrent use of voriconazole, a potent CYP3A4 inhibitor, may lead to increased plasma concentrations of ezetimibe, prolong its hepatic exposure, and elevate the metabolic burden on hepatocytes, thereby potentially inducing or exacerbating liver injury (LiverTox, 2012). Ezetimibe has been reported to cause severe cholestatic hepatitis, which resolved after drug discontinuation. Notably, the metabolite of ezetimibe, ezetimibe glucuronide, is a substrate of multidrug resistance-associated protein 2, the primary canalicular transporter responsible for biliary excretion of conjugated bilirubin from hepatocytes. Ezetimibe glucuronide competes with bilirubin for multidrug resistance-associated protein 2-mediated transport, thereby impairing bilirubin excretion and leading to isolated hyperbilirubinemia (Ritchie et al., 2008).

Prior studies have suggested that a dosage of 150 mg/day of caspofungin is associated with a lower risk of hepatic dysfunction (12%), making it a preferred option for treating invasive candidiasis (Domingos et al., 2022). However, the high incidence of hepatotoxicity observed in our cohort may indicate synergistic toxicity between caspofungin and voriconazole, necessitating further mechanistic studies to validate this hypothesis.

This retrospective study included 387 patients (50.4%) from the hematology department, where voriconazole was primarily administered for the prophylaxis of IFD in high-risk patients undergoing allogeneic hematopoietic stem cell transplantation. Glucocorticoids (GCs), commonly utilized as adjunctive therapy for hematologic malignancies, may correlate with an increased risk of DILI. Previous studies indicate that GCs, particularly dexamethasone, significantly reduce voriconazole plasma concentrations, with dexamethasone exhibiting the most pronounced effect in lowering voriconazole levels and increasing the incidence of subtherapeutic concentrations (Imataki et al., 2018; Jia et al., 2021). As potent CYP3A4 inducers, GCs may accelerate voriconazole metabolism, resulting in diminished plasma concentrations. Clinicians may need to escalate voriconazole doses to maintain therapeutic efficacy; however, inter-individual variability (e.g., CYP2C19 polymorphisms) could lead to drug accumulation and heightened hepatotoxicity in certain patients. Enhanced voriconazole metabolism via alternative pathways (e.g., CYP2C9) may produce hepatotoxic metabolites, such as voriconazole N-oxide, which can directly harm hepatocytes.

Prolonged or high-dose GC therapy may exacerbate liver injury through mechanisms such as steatosis, which occurs via lipolysis and the deposition of free fatty acids (Marino et al., 2016) or through mitochondrial dysfunction (Hernández-Alvarez et al., 2013). Although GCs exhibit weak intrinsic hepatotoxicity, their combination with Voriconazole may produce synergistic effects. Voriconazole induces ROS-mediated oxidative stress, while GCs may suppress antioxidant defenses, such as superoxide dismutase, thereby collectively amplifying hepatocellular damage (Luan et al., 2019).

This study reveals a significant inverse association between β-blocker use and the risk of voriconazole-induced hepatotoxicity (OR = 0.295, *P* = 0.032). Preclinical studies have demonstrated that carvedilol (CVL), a non-selective β-blocker, exerts hepatoprotective effects in toxin-induced liver injury models, independent of its β-adrenergic blocking properties (Wong et al., 2018). Specifically, CVL attenuates hepatic injury and ibrogenesis in a murine model of non-alcoholic fatty liver disease by suppressing ROS-dependent NOD-like receptor family, pyrin domain containing 3 inflammasome activation (Wong et al., 2018; Yu et al., 2022). Additionally, CVL downregulates inflammatory cytokine signaling in Kupffer cells and hepatic stellate cells, thereby mitigating oxidative stress, inflammation, and fibrosis in ethanol-induced liver injury models (Araújo et al., 2016). Furthermore, β-blockers have been shown to improve hepatic sinusoidal endothelial dysfunction in patients with cirrhosis by reducing portal pressure, alleviating sinusoidal congestion, and enhancing endothelial hypoxia resistance (Wu et al., 2019).Although β-blockers have demonstrated efficacy in reducing oxidative stress markers in models of alcoholic liver disease and non-alcoholic steatohepatitis, their protective role against DILI remains insufficiently explored. Further studies are necessary to validate the effects of β-blockers on oxidative stress, inflammatory responses, and sinusoidal hemodynamics in voriconazole-induced hepatotoxicity using preclinical animal models. Additionally, analysis of clinical cohort data is essential to confirm the observed inverse association between β-blocker use and voriconazole-related liver injury.

Thus, polypharmacy, especially with medications that are metabolized by the liver, represents an independent risk factor for DILI. When implementing combination therapy regimens, it is imperative to thoroughly evaluate the potential hepatotoxicity of each agent, as well as the possible drug-drug interactions and pharmacokinetic dynamics, to ensure both therapeutic safety and efficacy.

This study developed a nomogram prediction model for voriconazole-induced DILI by identifying key predictors through binary logistic regression analysis. The model demonstrated predictive accuracy, with AUC values of 0.728 and 0.773 for the training and test sets. Calibration curves revealed minimal deviation between predicted and observed results, with mean absolute errors of 0.01 and 0.018, respectively. DCA further confirmed the clinical utility of the model, showing substantial net benefits across a wide range of threshold probabilities. This study was the first to identify elevated TC levels as a novel predictor of voriconazole-induced hepatotoxicity. By integrating patient-specific characteristics, the nomogram provides actionable insights for clinicians to stratify DILI risk and optimize therapeutic decisions, such as initiating targeted monitoring or adjusting treatment regimens. However, this study has several limitations. First, trough plasma concentrations of voriconazole, which have been strongly linked to hepatotoxicity in prior studies, were excluded as a predictor due to insufficient therapeutic drug monitoring data at our institution. Incorporating trough plasma concentrations in future research could enhance the model’s predictive accuracy. Additionally, ALP, a key biomarker for cholestatic liver injury, was not included in the analysis because over 50% of the retrospective records lacked ALP measurements. Furthermore, this single-center, retrospective study included a limited number of ezetimibe-exposed cases (n = 7, <1%), representing a rare exposure. Although ezetimibe was associated with a significantly increased risk (adjusted OR = 7.45, *P* = 0.047) in the multivariate model, such rare events are susceptible to selection bias. Future multicenter studies with expanded sample sizes are warranted to validate the association between ezetimibe and voriconazole-associated DILI and to further explore its dose-response relationship. Finally, the impact of genetic polymorphisms in CYP2C19 and CYP3A4, critical enzymes governing voriconazole metabolism, could not be assessed due to the unavailability of genotypic data. These polymorphisms may contribute to inter-individual variability in hepatotoxicity risk by altering drug clearance and metabolite profiles.

## 5 Conclusion

In summary, the concomitant use of ezetimibe, glucocorticoids, or caspofungin is significantly associated with an increased risk of voriconazole-induced DILI. Based on these identified risk factors, we developed a nomogram prediction model for voriconazole-related DILI. This model facilitates the early identification of high-risk patients, enabling personalized treatment strategies to mitigate hepatotoxicity and reduce the incidence of voriconazole-related adverse effects.

## Data Availability

The original contributions presented in the study are included in the article/Supplementary Material, further inquiries can be directed to the corresponding authors.
