# Correction: Voriconazole-associated liver injury: clinical risk factor identification and predictive nomogram construction

**DOI:** 10.3389/fphar.2026.1834410

**Published:** 2026-03-27

**Authors:** Dongge Feng, Yiyi Ma, Xiaoyi Liu, Si Tang, Shijian Xiang, Jinghao Wang, Shasha Li

**Affiliations:** 1 Department of Pharmacy, The First Affiliated Hospital of Jinan University, Guangzhou, China; 2 School of Pharmacy, Jinan University, Guangzhou, China; 3 Department of Pharmacy, The Seventh Affiliated Hospital, Sun Yat-sen University, Shenzhen, China

**Keywords:** voriconazole, nomogram, drug-induced liver injury, predictive model, real-world study

An **Incorrect grant number** was provided for funder Science and Technology Projects in Guangzhou. The correct number is 2024A04J4104.

The original article has been updated.

