# Supplementary material for: Voriconazole-associated liver injury: clinical risk factor identification and predictive nomogram construction
Source: Front Pharmacol. 2026 Jan 5;16:1688711. doi: 10.3389/fphar.2025.1688711 (PMC12813146; doi:10.3389/fphar.2025.1688711)
Supplement: Supplementary file 1 [file Table1.docx]

**Table S1 Demographics, clinical characteristics and voriconazole therapy in 768 patients**

| Characteristic |  | Value |
| --- | --- | --- |
| Hepatotoxicity |  | 95 (12.4) |
| Gender | Male | 487 (63.4) |
| Age |  | 63 (49,73) |
| Treatment duration |  | 8 (4,15) |
| Days of hospitalization |  | 24 (14,37) |
| Department | Department of Respiratory Medicine | 120 (15.7) |
|  | Hematology | 387 (50.4) |
|  | Oncology | 13 (1.7) |
|  | ICU | 108 (14.1) |
|  | Infectious Diseases Department | 17 (2.2) |
|  | Hepatobiliary Medicine | 1 (0.1) |
|  | Nephrology | 39 (5.1) |
|  | Endocrinology | 7 (0.9) |
|  | Urology | 22 (2.9) |
|  | Gastrointestinal Surgery | 5 (0.7) |
|  | Emergency Department | 2 (0.3) |
|  | Rheumatology | 3 (0.4) |
|  | Thoracic Surgery | 3 (0.4) |
|  | Gynecology | 1 (0.1) |
|  | Neurology | 13 (1.7) |
|  | Breast Surgery Ward | 1 (0.1) |
|  | Cardiology | 19 (2.5) |
|  | Spine and Trauma Department | 3 (0.4) |
|  | Burn and Plastic Surgery Department | 1 (0.1) |
|  | General Medicine Department | 1 (0.1) |
|  | Gastroenterology Department | 1 (0.1) |
|  | Ophthalmology Department | 1 (0.1) |
| Dosage forms | Tablet | 484 (63.02) |
|  | Injection | 225 (29.30) |
|  | Injection and Tablet | 58 (7.55) |
|  | eye drops | 1 (0.13) |
| Alcohol |  | 39 (5.1) |
| Smoking |  | 91 (11.8) |
| complications | Hypoproteinemia | 228 (29.7) |
|  | Malignant Tumor | 80 (10.4) |
|  | Hematologic Malignancies | 301 (39.2) |
|  | Septic Shock | 86 (11.2) |
|  | Hyperuricemia | 52 (6.8) |
|  | Anemia | 87 (11.3) |
|  | Sepsis | 68 (8.9) |
|  | Cardiovascular Diseases | 71 (9.2) |
|  | CKD | 218 (28.4) |
|  | Pulmonary Infection | 346 (45.1) |
|  | Heart Disease | 213 (27.7) |
|  | COPD | 34 (4.4) |
|  | Hypertension | 241 (31.4) |
|  | Diabetes Mellitus | 146 (19.0) |
| Purpose of medication | Treatment | 415 (54) |
|  | Prophylaxis | 273 (35.5) |
|  | Empirical | 80 (10.4) |
| Site of infection | Respiratory Tract Infection | 365 (47.5) |
|  | Urinary System | 38 (4.9) |
|  | Central Nervous System Infection | 5 (0.7) |
|  | Intestinal Infection | 2 (0.3) |
|  | Intra-abdominal Infection | 8 (1.0) |
|  | Skin Infection | 5 (0.7) |
| Type of pathogen | *Aspergillus* | 152 (19.8) |
|  | *Candida* | 122 (15.9) |
|  | Other Fungi | 63 (8.2) |
| Clinical outcome | Improvement | 508 (66.1) |
|  | Inefficacy | 90 (11.7) |
|  | Mortality | 67 (8.7) |
| Adverse Reactions | Neurotoxicity | 25 (3.3) |
|  | Ocular Toxicity | 9 (1.2) |
|  | Other Adverse Reactions | 24 (3.1) |
| Adverse Drug Reaction | Occurrence | 213 (27.7) |
| Hepatotoxicity | Grade 1:2:3:4 | 44:30:18:3 |
| Liver Injury Clinical Types | Hepatocellular Injury | 33 (34.7) |
|  | Cholestatic Injury | 42 (44.2) |
|  | Mixed Pattern Liver Injury | 20 (21.1) |
| Time of adverse reaction onset |  | 4 (2,8) |
| Meet the criteria of DILI | RUCAM score ≥ 6 | 95 (12.4) |
|  | RUCAM score 3-5 | 60 (7.8) |

Data are presented as number of patients (%) or median (interquartile range). DILI, drug-induced liver injury; ICU, intensive care unit; CKD, Chronic Kidney Disease; COPD, Chronic Obstructive Pulmonary Disease.

**Table S2. The risk variables selected by Lasso model**

| variable | coef |
| --- | --- |
| Septic shock | 0.00000000 |
| sulfamethoxazole | 0.03824002 |
| Caspofungin | 0.16666988 |
| Glucocorticoids | 0.21742861 |
| β-adrenergic antagonist | -0.33323510 |
| Rabeprazole | 0.00000000 |
| Omeprazole | 0.00000000 |
| Bromhexine | 0.04042454 |
| Salbutamol | 0.00000000 |
| Montelukast sodium | 0.05315561 |
| Terbutaline | 0.07098134 |
| Ezetimibe | 0.13930071 |
| PCT (ng/mL) | 0.02157760 |
| WBC (×10⁹/L) | 0.06281571 |
| TC (mmol/L) | 0.78108451 |
